# Activities of cardiac tissue matrix metalloproteinases 2 and 9 are reduced by remote ischemic preconditioning in cardiosurgical patients with cardiopulmonary bypass

**DOI:** 10.1186/1479-5876-12-94

**Published:** 2014-04-08

**Authors:** Karina Zitta, Patrick Meybohm, Berthold Bein, Matthias Gruenewald, Fabian Lauer, Markus Steinfath, Jochen Cremer, Kai Zacharowski, Martin Albrecht

**Affiliations:** 1 Department of Anaesthesiology and Intensive Care Medicine, University Hospital Schleswig-Holstein, Kiel, Germany; 2 Clinic of Anaesthesiology, Intensive Care Medicine and Pain Therapy, University Hospital Frankfurt, Frankfurt am Main, Germany; 3 Department of Cardiovascular Surgery, University Hospital Schleswig-Holstein, Kiel, Germany

**Keywords:** Cardioprotection, Ischemia/reperfusion injury, Matrix metalloproteinases, Myocardial damage, Remote ischemic preconditioning

## Abstract

**Background:**

Transient episodes of ischemia in a remote organ or tissue (remote ischemic preconditioning, RIPC) can attenuate myocardial injury. Myocardial damage is associated with tissue remodeling and the matrix metalloproteinases 2 and 9 (MMP-2/9) are crucially involved in these events. Here we investigated the effects of RIPC on the activities of heart tissue MMP-2/9 and their correlation with serum concentrations of cardiac troponin T (cTnT), a marker for myocardial damage.

**Methods:**

In cardiosurgical patients with cardiopulmonary bypass (CPB) RIPC was induced by four 5 minute cycles of upper limb ischemia/reperfusion. Cardiac tissue was obtained before as well as after CPB and serum cTnT concentrations were measured. Tissue derived from control patients (N = 17) with high cTnT concentrations (≥0.32 ng/ml) and RIPC patients (N = 18) with low cTnT (≤0.32 ng/ml) was subjected to gelatin zymography to quantify MMP-2/9 activities.

**Results:**

In cardiac biopsies obtained before CPB, activities of MMP-2/9 were attenuated in the RIPC group (MMP-2: Control, 1.13 ± 0.13 a.u.; RIPC, 0.71 ± 0.12 a.u.; P < 0.05. MMP-9: Control, 1.50 ± 0.16 a.u.; RIPC, 0.87 ± 0.14 a.u.; P < 0.01), while activities of the pro-MMPs were not altered (P > 0.05). In cardiac biopsies taken after CPB activities of pro- and active MMP-2/9 were not different between the groups (P > 0.05). Spearman’s rank tests showed that MMP-2/9 activities in cardiac tissue obtained before CPB were positively correlated with postoperative cTnT serum levels (MMP-2, P = 0.016; MMP-9, P = 0.015).

**Conclusions:**

Activities of MMP-2/9 in cardiac tissue obtained before CPB are attenuated by RIPC and are positively correlated with serum concentrations of cTnT. MMPs may represent potential targets for RIPC mediated cardioprotection.

**Trial registration:**

ClinicalTrials.gov identifier NCT00877305.

## Background

Cardiac surgery with cardiopulmonary bypass is generally associated with a predictable incidence of myocardial, neurological, and renal ischemia/reperfusion injury leading to an increased risk of post-operative myocardial stunning, neurological deficits, acute renal failure and as a result increased mortality [1-3].

Ischemic preconditioning in which transient episodes of ischemia are applied before prolonged ischemia/reperfusion injury has been shown to reduce myocardial damage resulting in cardioprotection [4-8]. Ischemic preconditioning does not only act locally, but also protects remote tissues from ischemia/reperfusion injury, a phenomenon known as remote ischemic preconditioning (RIPC). Studies in patients reported that transient limb ischemia attenuates myocardial injury in a number of clinical situations, including coronary artery surgery, congenital heart surgery, and non-cardiac surgery of high-risk patients [6,8-15]. In our recent study we investigated cellular and molecular effects of RIPC in heart tissue of cardiosurgical patients with cardiopulmonary bypass (CPB) and showed that RIPC regulates HIF-1α levels, apoptosis and inflammation [16]. The clinical outcome of ischemia/reperfusion injury in the heart is also strongly dependent on remodeling processes within the myocardial tissue. Matrix metalloproteinases (MMPs), are members of the metzincin group of proteases, which are named after the zinc ion and the conserved Met residue at the active site [17] and especially MMP-2 and MMP-9 are believed to play a key role in remodeling processes within the myocardial tissue [18,19]. Besides their involvement in tissue remodeling, various other biological consequences are also based on the proteolytic activities or MMPs: MMPs regulate several chemokines and affect cell survival as well as cell proliferation. Moreover, MMPs induce cell differentiation and are also able to activate latent signaling molecules or inactivate soluble mediators [20]. Based on their multiple functions, MMPs may therefore represent so far neglected cellular targets for RIPC-mediated cardioprotection.

In the study presented, we investigated the effect of RIPC on the activities of MMP-2 and MMP-9 in cardiac biopsies obtained from cardiosurgical patients before and after CPB and screened for a possible correlation of activities of cardiac tissue MMP-2/9 and postoperative serum cTnT concentrations.

## Methods

### Experimental protocol

**Figure 1:**
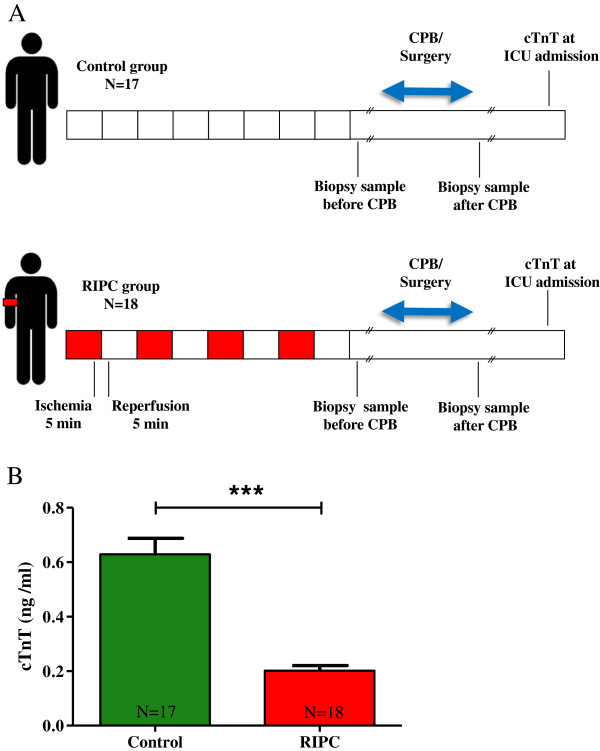
**Experimental setting and serum cardiac troponin T (cTnT) concentrations in control and RIPC patients.** RIPC was performed by 4 cycles of 5 minutes of upper arm ischemia induced with a blood pressure cuff. Each cycle of ischemia was followed by 5 minutes of reperfusion. Cardiac biopsies were obtained before and after CPB **(A)**. cTnT levels were evaluated at ICU admission in the control and RIPC group **(B)**. Bars denote SEM; ***, P < 0.001. cTnT, cardiac troponin T; CPB, cardiopulmonary bypass; ICU, intensive care unit; RIPC, remote ischemic preconditioning.

The study protocol, patient information, and informed consent were approved by the Ethics Committee of the University Hospital Schleswig-Holstein, Campus Kiel, Germany (Reference number: A165/08). The study was performed in accordance with the fourth revision of the Declaration of Helsinki (1996) and is registered at ClinicalTrials.gov (NCT00877305). Employing patient sera and biopsy material an experimental substudy has been published recently [16] and clinical data focusing on neurocognitive outcome have been presented by Meybohm et al [21]. Aim of the actual study was to investigate a possible involvement of MMP-2/9 activity in RIPC-mediated cardioprotection and patients included into the study were selected based on blood levels of cardiac troponin T (cTnT; for details see below). Each patient (age ≥ 18 years) gave written informed consent to participate in the study. All types of cardiac surgery in which cardiopulmonary bypass (CPB) was used were included. Patients were randomized to group RIPC or control in a double-blinded fashion. RIPC was induced by four cycles of upper limb ischemia (5-minutes blood-pressure cuff inflation to 200 mmHg and 5-minutes cuff deflation) after induction of total intravenous anaesthesia (propofol and sufentanil). RIPC treatment was predominantly assigned to the left upper arm. In patients assigned to the control group we used four cycles of 5-minutes blood-pressure cuff inflation to 20 mmHg and 5-minutes cuff deflation without any limb ischemia (Figure 1A). Basic and clinical information about the respective patients included in the experiments are displayed in Tables 1 and 2.

**Table 1:** Basic and clinical data of RIPC and control patients

**Variable**	**Control (N = 17)**	**RIPC (N = 18)**	**P-value**
Demographic data			
Male (N)	17 (100%)	14 (77.8%)	0.10
Female (N)	0 (0%)	4 (22.2%)	0.10
Age (years)	63 ± 9	68 ± 11	0.03
Weight (kg)	86 ± 9.5	80 ± 11	0.04
BMI (kg/m2)	28 ± 3.2	26 ± 3.6	0.04
Type of surgery			
CABG (N)	9 (52.9%)	13 (72.2%)	0.31
AVR (N)	2 (11.8%)	2 (11.1%)	1.00
Combi (N)	6 (35.3%)	3 (16.7%)	0.26
Perioperative data			
Bypass time (min)	128 ± 34	108 ± 51	0.01
Aortic clamping time (min)	86 ± 25	70 ± 36	0.01
EuroSCORE	3 ± 2	4 ± 2	0.03

AVR, aortic valve replacement; BMI, body mass index; CABG, coronary artery bypass graft; Combi, combined surgery.

**Table 2:** Comorbidities, medications and ICU data of RIPC and control patients

**Variable**	**Control (N = 17)**	**RIPC (N = 18)**	**P-value**
Comorbidities			
Diabetes mellitus (N)	3 (17.6%)	4 (22.2%)	1.00
Hypercholesterolemia (N)	12 (70.6%)	9 (50.0%)	0.31
Stroke (N)	2 (11.8%)	1 (5.5%)	0.60
Arterial hypertension (N)	14 (82.3%)	15 (83.3)	1.00
Myocardial infarction (N)	3 (17.6%)	7 (38.8%)	0.26
Medications			
Beta-adrenergic-blockers (N)	10 (58.8%)	13 (72.2%)	0.49
ACE/AT-1-antagonists (N)	11 (64.7%)	8 (44.4%)	0.31
ASA/clopidogrel (N)	14 (82.3%)	13 (72.2%)	0.69
Calcium-channel blockers (N)	1 (5.9%)	3 (16.6%)	0.60
Cholesterol-lowering (N)	8 (47.1%)	9 (50.0%)	1.00
Insulin/metformin (N)	2 (11.8%)	3 (16.6%)	1.00
Diuretics (N)	5 (29.4%)	3 (16.6%)	0.44
ICU data			
ICU-stay (hours)	59 ± 57	28 ± 23	0.13
ICU-readmission (N)	0 (0%)	1 (5.5%)	1.00
Reoperation (N)	1 (5.9%)	0 (0%)	0.49
Arrythmia (N)	4 (23.5%)	7 (38.8%)	0.47
Ventilation time (hours)	26 ± 25	14 ± 5	0.15
Reintubation (N)	3 (17.6%)	1 (5.5%)	0.34

### Tissue and serum samples

Cardiac tissue of the right atrium was collected from RIPC and control patients before as well as after CPB. cTnT values were obtained for each patient at the following time points: (i) before surgery, (ii) at intensive care unit (ICU) admission, (iii) after 12 h, (iv) after 24 h, and (v) after 48 h. As in our previous study statistically significant differences in cTnT concentrations between control and RIPC patients were only detected at ICU admission [16], all further analyses were performed with cTnT data from this time point. Quantifications of cTnT were performed by the Department of Clinical Chemistry of the University Hospital Schleswig-Holstein, Campus Kiel, Germany. All tissue und serum samples analyzed in the study were derived from the collective of the patients employed in our recently published work [16]. The mean concentration of cTnT in the patients investigated was 0.42 ± 0.05 ng/ml (median: 0.32 ng/ml [0.19/0.57]; [1^st^/3^rd^] percentile). In order to optimize the experimental setup for the analyses of a possible involvement of MMP-2/9 in RIPC-mediated cardioprotection (classified by cTnT levels), we established subgroups of patients based on blood concentrations of cTnT. Only cardiac tissue from control patients with high cTnT concentrations (≥0.32 ng/ml at ICU admission) indicative of myocardial damage [22] and from RIPC patients with low cTnT concentrations, indicative of cardioprotection ("responder"; ≤0.32 ng/ml at ICU admission) was included into the study.

### Gelatin zymography

Protein extraction from tissue biopsies was performed with RIPA buffer containing 150 mM sodium chloride, 1.0% NP-40, 0.1% sodium dodecyl sulfate (SDS), 1% sodium deoxycholate, 50 mM Tris-HCl (pH 7.6; all from Sigma-Aldrich, Hamburg, Germany). Protein concentrations were determined with a BCA Protein Assay kit (RotiQ, Carl Roth, Karlsruhe, Germany). Zymography was performed as described previously [23,24]. Briefly, 30 μg of the respective tissue was loaded and separated on 7% SDS polyacrylamide gels (containing 1 mg/ml gelatin) under non-reducing conditions. After electrophoresis the gels were soaked in 2.5% Triton X-100 for 30 minutes to remove SDS and incubated in Tris-HCl (50 mmol/l, pH 7.5), containing CaCl_2_ (5 mmol/l), and ZnCl_2_ (1 mmol/l) overnight at 37 °C. After Coomassie blue staining white bands indicated digestion of gelatin by MMPs. Densitometric analysis was performed using the ImageJ 1.41o software (ImageJ, NIH, USA). As even slight differences in the treatment procedure of the gelatin gels (e.g. incubation time, staining and destaining protocols) resulted in variations in the signal intensities between the respective gels, a control of human recombinant MMP-2 (0.1 ng; #PF023, Merck, Darmstadt, Germany) was loaded on each gel and signal intensities of the MMP bands were related to the signal intensity of 0.1 ng MMP-2 on the respective gel.

### Statistical analysis

Statistics were performed using the software GraphPad Prism version 5.01 for Windows. Categorical variables were compared using the Fisher’s exact test. Each data set was tested for normality using the D’Agostino and Pearson omnibus test and student’s t-tests were performed. For MMP-2/9 and cTnT correlations, the Spearman’s rank correlation coefficient [25,26] was employed. Variables are expressed as mean ± SEM.

## Results

### Serum concentrations of cTnT and clinical parameters of RIPC patients

cTnT concentrations are elevated in the blood after myocardial tissue damage [22,27,28]. In a study including 61 patients we showed that RIPC significantly reduced cTnT levels in the blood of cardiosurgical patients directly after admission to the intensive care unit (ICU; control, 0.60 ± 0.06 ng/ml; RIPC, 0.45 ± 0.06 ng/ml; P < 0.05; [16]). However, cTnT levels were only by trend reduced by RIPC in our recent study which included a total of 90 control and 90 RIPC patients [21]. Here, 17 control patients with mean cTnT concentrations of 0.63 ± 0.06 ng/ml and 18 RIPC patients with mean cTnT concentrations of 0.20 ± 0.02 ng/ml (P < 0.001, Figure 1B) were included into the study and subjected to further analyses. In addition to the significant reduction of cTnT concentrations in the RIPC group, RIPC patients also exhibited a by trend shorter mean residence time at the ICU (control, 59 ± 57 h; RIPC, 28 ± 23 h; Table 2) which was accompanied by a reduced ventilation time (control, 26 ± 25 h; RIPC, 14 ± 5 h; Table 2).

### MMP-2 and MMP-9 activities in cardiac tissue obtained before CPB are attenuated in RIPC patients

**Figure 2:**
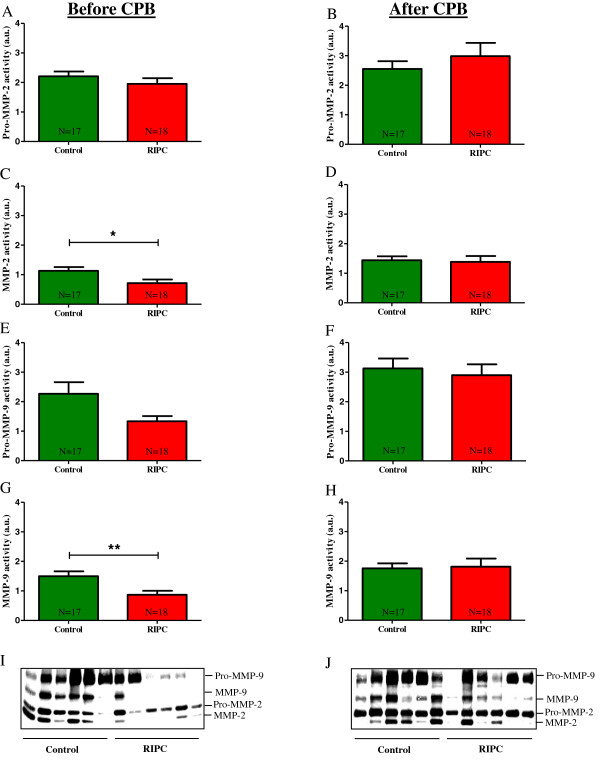
**MMP activities in cardiac biopsy samples.** The enzymatic activities of pro-MMP-2, MMP-2, pro-MMP-9 and MMP-9 were evaluated in tissue samples taken before CPB **(A, C, E, G)** and after CPB **(B, D, F, H)** using gelatin zymography. One representative gel containing 6 control and 6 RIPC samples is shown in **I** (before CPB) and **J** (after CPB). Bars denote SEM; *, P < 0.05; **, P < 0.01. CPB, cardiopulmonary bypass; RIPC, remote ischemic preconditioning.

Biological consequences of increased or decreased MMP activities are complex and include tissue remodeling, cell proliferation, cell death, inactivation of soluble mediators and activation of latent signaling molecules [20]. We show that in cardiac biopsies taken before CPB, activities of pro-MMP-2 and pro-MMP-9 were not altered (P > 0.05; Figure 2 A, E, I) while the activities of MMP-2 and MMP-9 were significantly lower in RIPC patients compared to the control group (MMP-2: RIPC, 0.71 ± 0.12 a.u.; Control, 1.13 ± 0.13 a.u.; P < 0.05. MMP-9: RIPC, 0.87 ± 0.14 a.u.; Control, 1.50 ± 0.16 a.u.; P < 0.01; Figure 2 C, G, I). In cardiac biopsy samples that were obtained after CPB, activities of pro- and active MMP-2 and MMP-9 were not different between the control and RIPC group (P > 0.05; Figure 2 B, D, F, H, J). Additional control experiments were performed using cardiac tissue samples from RIPC patients with high cTnT levels ("non-responder"; cTnT ≥0.32 ng/ml; mean cTnT concentration 1.04 ± 0.14 ng/ml; N = 12) and control patients with low cTnT blood concentrations (cTnT ≤0.32 ng/ml; mean cTnT concentration 0.18 ± 0.02 ng/ml; N = 12). The results show an opposite trend in the correlation between RIPC and MMP-2/9 activity. Compared to the control group, the activities of MMP-2 and MMP-9 in cardiac tissue obtained before CPB were by trend increased in the RIPC group (Additional file 1: Figure S1). To investigate whether the effects of RIPC on MMP-2/9 activities are mediated via the regulation of protein expression levels, Westernblotting experiments were performed and revealed that RIPC did not change the relative amounts of MMP-2 and MMP-9 protein in cardiac tissue derived before or after CPB (Additional file 2: Figure S2).

### Serum concentrations of cTnT are positively correlated with MMP-2 and MMP-9 activities in cardiac tissue obtained before CPB

**Figure 3:**
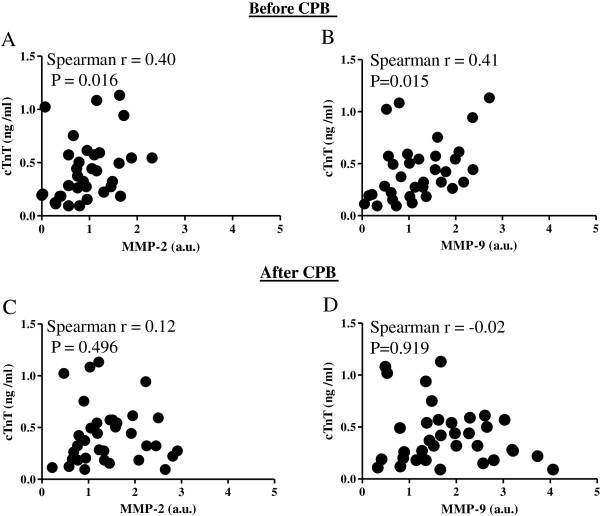
**Correlation of cardiac tissue MMP-2/9 activities and serum cardiac troponin T (cTnT) levels.** cTnT concentrations were measured at admission to the intensive care unit (ICU) and were correlated with the activities of MMP-2 and MMP-9 in heart tissue samples obtained before CPB **(A, B)** and after CPB **(C, D)**. CPB, cardiopulmonary bypass; cTnT, cardiac troponin T.

As RIPC was related to decreased activities of MMP-2 and MMP-9 in cardiac biopsies obtained before CPB (Figure 2 C, G, I), we next investigated whether the activities of cardiac tissue MMP-2 and MMP-9 also correlated with postoperative serum concentrations of cTnT, a well-recognized marker of myocardial tissue damage [22,27,28]. Spearman’s rank tests [25,26] showed that MMP-2 and MMP-9 activities in cardiac tissue biopsies obtained before CPB were positively correlated with cTnT serum levels (MMP-2/cTnT: Spearman r, 0.40; P = 0.016. MMP-9/cTnT: Spearman r, 0.41; P = 0.015; Figure 3 A, B). No significant correlation between cTnT concentrations and MMP-2/9 activity was found in samples taken after CPB (MMP-2/cTnT: Spearman r, 0.12; P = 0.496. MMP-9/cTnT: Spearman r, -0.02; P = 0.919; Figure 3 C, D).

## Discussion

Remote ischemic preconditioning (RIPC) is a straightforward and efficient method to protect the heart from ischemia/reperfusion injury which appears during various surgical procedures [8,10,12,15,16,29,30]. Although, recent studies clearly showed the capacity of RIPC in the clinic, cellular and molecular mechanisms of RIPC within the heart are only poorly investigated [31,32]. Detailed information about the RIPC-mediated events and target molecules could however increase the effectiveness of RIPC leading to improved patient outcome.

Cardiac surgery with cardiopulmonary bypass (CPB) is frequently associated with ischemia/reperfusion injury leading to cell death and subsequent tissue remodeling [33,34]. MMP-2 and MMP-9 are believed to play a key role in the ischemia/reperfusion induced myocardial remodeling processes and may therefore represent potential targets for cardioprotective interventions such as RIPC [8,18,33,35]. Employing a pig model of cardiac arrest following left anterior descending coronary artery ischemia, we showed a significantly increased enzymatic activity of MMP-9 in the ischemic myocardial tissue [36]. Moreover, we recently demonstrated that RIPC reduces the activities of MMP-2 and MMP-9 in sera of cardiosurgical patients [24]. Here we reveal that tissue activities of MMP-2 and MMP-9 are also attenuated by RIPC in cardiac biopsies obtained before CPB. Our findings suggest a possible connection between MMP activities and ischemia/reperfusion induced cardiac damage, supporting the idea that low MMP-2/9 activities may be associated with reduced ischemia/reperfusion injury. How the ischemic preconditioning in a remote organ (upper limb) can lead to a reduction of MMP-2/9 activity in cardiac tissue is still unclear. In general, three main mechanisms may be responsible for transmitting the protective signal from the organ or tissue, in which the RIPC stimulus is applied, to the target organ or tissue: (i) Neural pathways. (ii) The release of circulating humoral factor(s). (iii) Activation of a systemic protective effect (e.g. anti-inflammatory response) [29]. It is not yet known if and how these pathways are interconnected and how they could influence the activities of tissue MMPs. However, MMP function can be regulated at many levels. In addition to RNA transcription and protein synthesis, MMP function can be controlled at the levels of secretion, intracellular trafficking, subcellular or extracellular localization, activation of the zymogen form, expression of MMP specific endogenous protein inhibitors, such as tissue inhibitors of metalloproteinases (TIMPs) and alpha2-macroglobulin, and protease degradation [20]. As in our study RIPC regulated enzymatic activities, but not protein levels of MMP-2/9, inhibitory molecules like TIMPs and alpha2-macroglobulin may be suggested as potential targets of the RIPC mediated attenuation of MMP activity. Interestingly, MMP-2/9 activities were not regulated by RIPC in tissue samples taken after CPB. This finding might be based on the fact that the immunological response to the extracorporeal circulation during CPB generates a systemic inflammatory response syndrome (SIRS), that is associated with the release of various pro-and anti-inflammatory cytokines [37,38] which may influence the activities of cardiac MMP-2/9, masking the RIPC-mediated effects in tissue obtained after CPB. On the other hand, there is also the possibility that RIPC-mediated effects appear very early after induction of RIPC and are of only short and transitory nature. It is known that ischemic preconditioning represents a biphasic phenomenon with a first and a second window of protection and similar mechanisms may also be effective in remote ischemic preconditioning. The early phase of protection develops quickly within minutes from the initial ischemic conditioning event and lasts for 2 to 3 hours. This is followed by a delayed phase that begins after 12 to 24 hours and lasts up to 4 days. In our study, intraoperative ischemia falls into the first window of protection. The mechanisms of the two phases of protection are believed to be of very different nature. While the early phase is caused by rapid modification and/or activation of pre-existing proteins, the delayed phase requires synthesis of new proteins. [39,40]. This may explain our observation that activities of MMP-2 and MMP-9 are regulated by RIPC, while we did not observe effects of RIPC on the protein levels of both MMPs. The fact that the first window of protection only lasts for 2-3 hours could also be responsible for our findings that changes in MMP activities were only evident shortly after RIPC, at the beginning of the surgical procedure but not anymore at the end of surgery.

Blood concentrations of cTnT are typically increased after myocardial injury [22,27,28]. In our previous study we showed that serum concentrations of cTnT are reduced in patients receiving RIPC [16] an observation that has also been made by others, pointing towards cardioprotective effects of RIPC [8,10,12,15,41]. In the study presented, we found a positive correlation of tissue MMP-2/9 activities in cardiac biopsies taken before CPB and serum levels of cTnT at ICU admission. These data once more support the idea that the RIPC-associated reduction of cardiac tissue MMP-2 and MMP-9 activities may be directly or indirectly involved in RIPC-mediated cardioprotection. Serum cTnT levels did not correlate with MMP-2/9 activities in cardiac tissue taken after CPB. This observation may be related to the SIRS generated by the extracorporeal circulation during CPB, a short and only transitory effect of RIPC on MMP-2/9 activities or other yet unidentified mechanisms induced by the surgical or anaesthetic procedure.

There are several limitations of the study, which have to be considered: (i) Due to surgical restrictions and refusal of several patients to the procedure of biopsy retrieval, cardiac tissue could not be obtained from all patients that participated in the initial study (ClinicalTrials.gov; NCT00877305). (ii) In the case that biopsies were taken from the respective patients, only a limited amount of tissue (<1 mm^2^) was available for biochemical analyses. (iii) Based on surgical limitations only cardiac tissue of the right atrium and no ventricular tissue could be obtained. (iv) The first biopsy sample was collected directly after RIPC (before CPB). As the second biopsy was collected immediately after CPB, the time period between the collection of the first and second sample varied between the patients and was determined by the length of the surgical procedure. (v) Although, there were no differences in co-morbidities, medications and ICU data between control and RIPC patients, some of the perioperative parameters differed between the groups. While bypass time and aortic clamping time were lower in RIPC patients possibly resulting in reduced perioperative stress and tissue damage, age, weight and EuroSCORE were all significantly higher in the RIPC group predisposing these patients to an increased peri- and postoperative myocardial damage. Moreover, while the control group consisted only of male patients (17/17), 22% of the patients in the RIPC group were female (4/18). The differences in gender distribution did not reach statistically significant levels, however we cannot exclude that the gender variations between the groups may have at least to some extend influenced the results of the study, especially as several clinical observations suggest increased resistance of the female heart to ischemia/reperfusion injury [15,42].

## Conclusions

Our study performed with tissue and serum samples from cardiosurgical patients showed that activities of MMP-2/9 in cardiac tissue obtained before CPB are attenuated by RIPC and are positively correlated with serum concentrations of cTnT, suggesting that MMP-2/9 may be involved in RIPC-mediated cardioprotection. However, additional studies have to clarify whether the observed reduction of cardiac MMP-2/9 activities by RIPC is causally related to RIPC-mediated cardioprotection and if a modulation of MMP-2/9 activities has the potential to augment the cardioprotective efficacy of RIPC in cardiosurgical patients.

## Competing interests

The authors declare that they have no competing interests.

## Authors’ contributions

PM, MG, JC and FL were involved in the acquisition and evaluation of the clinical data. KZi and PM performed the analyses of the tissue samples. MA was responsible for the study concept and design, as well as drafting of the manuscript. BB, MS and KZa were involved in the statistical analyses and critical revision of the manuscript for important intellectual content. All authors read and approved the final manuscript.

## Supplementary Material

Additional file 1: Figure S1 MMP activities in cardiac biopsy samples. The enzymatic activities of pro-MMP-2, MMP-2, pro-MMP-9 and MMP-9 were evaluated in tissue samples from 12 control patients with low cTnT levels (cTnT ≤0.32 ng/ml; mean cTnT concentration 0.18 ± 0.02 ng/ml) and 12 RIPC patients with high cTnT concentrations ("non-responder"; cTnT ≥0.32 ng/ml; mean cTnT concentration 1.04 ± 0.14 ng/ml). Compared to the control group the activities of both MMPs were by trend increased in the RIPC group with high cTnT levels. Bars denote SEM. CPB, cardiopulmonary bypass; RIPC, remote ischemic preconditioning. Click here for file

Additional file 2: Figure S2 Proteinexpression of MMP-2/9. Westernblotting experiments were performed using cardiac tissue derived before CPB (A, C) and after CPB (B, D). Antibodies against MMP-2 (A, B) and MMP-9 (C, D) were employed and the intensity of the respective signal was related to the intensity of actin. One representative Westernblot containing samples from 6 control and 6 RIPC patients is shown in E (before CPB) and F (after CPB). Bars denote SEM. Click here for file
