# Supplementary material for: Activities of cardiac tissue matrix metalloproteinases 2 and 9 are reduced by remote ischemic preconditioning in cardiosurgical patients with cardiopulmonary bypass
Source: J Transl Med. 2014 Apr 8;12:94. doi: 10.1186/1479-5876-12-94 (PMC4234318; doi:10.1186/1479-5876-12-94)
Supplement: Additional file 1: Figure S1 — MMP activities in cardiac biopsy samples. The enzymatic activities of pro-MMP-2, MMP-2, pro-MMP-9 and MMP-9 were evaluated in tissue samples from 12 control patients with low cTnT levels (cTnT ≤0.32 ng/ml; mean cTnT concentration 0.18 ± 0.02 ng/ml) and 12 RIPC patients with high cTnT concentrations ("non-responder"; cTnT ≥0.32 ng/ml; mean cTnT concentration 1.04 ± 0.14 ng/ml). Compared to the control group the activities of both MMPs were by trend increased in the RIPC group with high cTnT levels. Bars denote SEM. CPB, cardiopulmonary bypass; RIPC, remote ischemic preconditioning. [file 1479-5876-12-94-S1.ppt]

## Slide 1
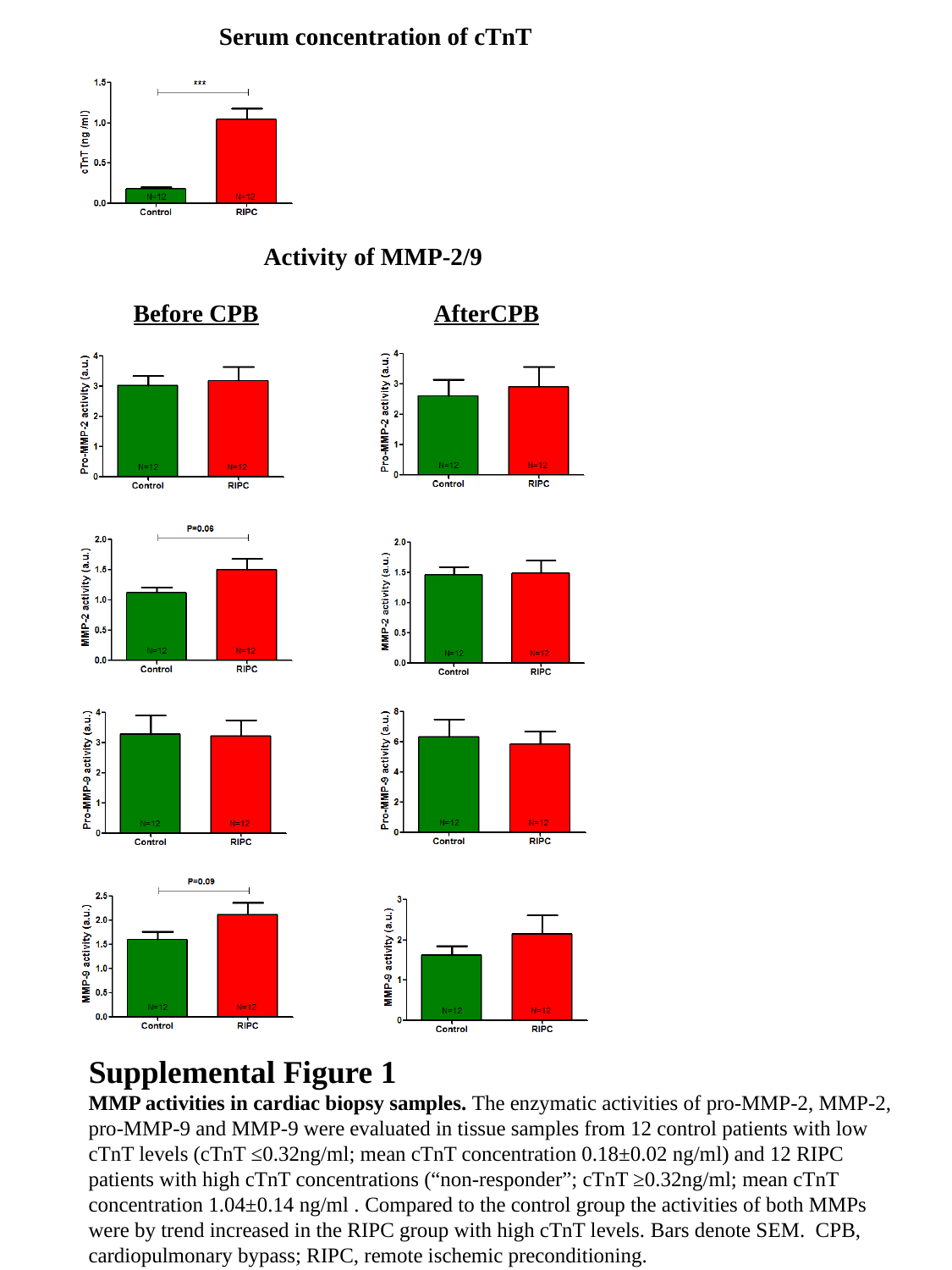

Serum concentration of cTnT
Activity of MMP-2/9
Before CPB
AfterCPB
Supplemental Figure 1
MMP activities in cardiac biopsy samples. The enzymatic activities of pro-MMP-2, MMP-2, pro-MMP-9 and MMP-9 were evaluated in tissue samples from 12 control patients with low cTnT levels (cTnT ≤0.32ng/ml; mean cTnT concentration 0.18±0.02 ng/ml) and 12 RIPC patients with high cTnT concentrations (“non-responder”; cTnT ≥0.32ng/ml; mean cTnT concentration 1.04±0.14 ng/ml . Compared to the control group the activities of both MMPs were by trend increased in the RIPC group with high cTnT levels. Bars denote SEM. CPB, cardiopulmonary bypass; RIPC, remote ischemic preconditioning.
