# Supplementary material for: Activities of cardiac tissue matrix metalloproteinases 2 and 9 are reduced by remote ischemic preconditioning in cardiosurgical patients with cardiopulmonary bypass
Source: J Transl Med. 2014 Apr 8;12:94. doi: 10.1186/1479-5876-12-94 (PMC4234318; doi:10.1186/1479-5876-12-94)
Supplement: Additional file 2: Figure S2 — Proteinexpression of MMP-2/9. Westernblotting experiments were performed using cardiac tissue derived before CPB (A, C) and after CPB (B, D). Antibodies against MMP-2 (A, B) and MMP-9 (C, D) were employed and the intensity of the respective signal was related to the intensity of actin. One representative Westernblot containing samples from 6 control and 6 RIPC patients is shown in E (before CPB) and F (after CPB). Bars denote SEM. [file 1479-5876-12-94-S2.ppt]

## Slide 1
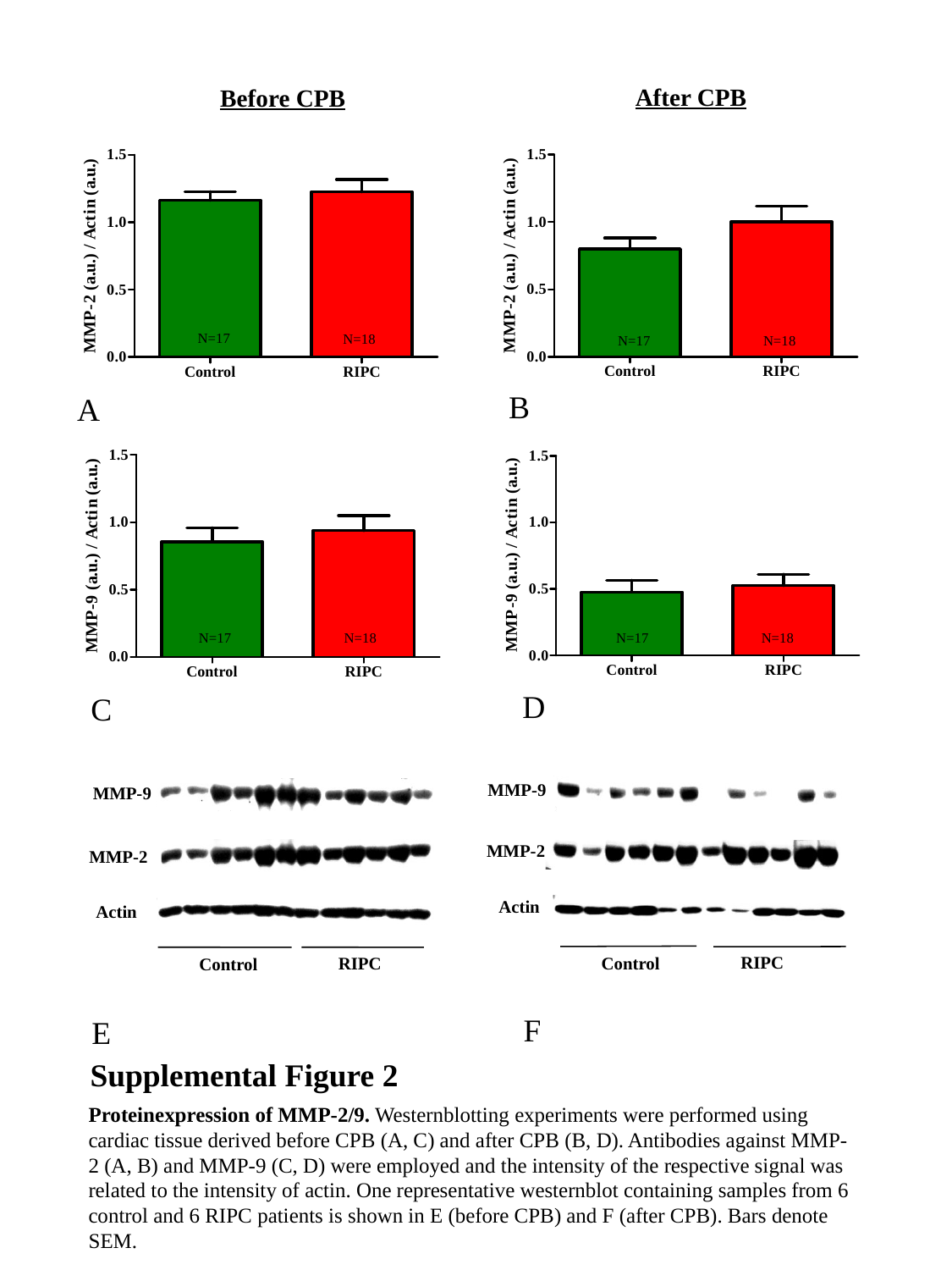

After CPB
Before CPB
N=17
N=18
N=17
N=18
B
A
N=17
N=18
N=17
N=18
D
C
MMP-9
MMP-9
MMP-2
MMP-2
Actin
Actin
RIPC
RIPC
Control
Control
F
E
Supplemental Figure 2
Proteinexpression of MMP-2/9. Westernblotting experiments were performed using cardiac tissue derived before CPB (A, C) and after CPB (B, D). Antibodies against MMP-2 (A, B) and MMP-9 (C, D) were employed and the intensity of the respective signal was related to the intensity of actin. One representative westernblot containing samples from 6 control and 6 RIPC patients is shown in E (before CPB) and F (after CPB). Bars denote SEM.
